# The Efficacy of Disability Employment Service (DES) Providers Working with Autistic Clients

**DOI:** 10.1007/s10803-022-05762-1

**Published:** 2022-09-28

**Authors:** Susan M. Hayward, Rebecca L. Flower, Kathleen E. Denney, Simon Bury, Amanda L. Richdale, Cheryl Dissanayake, Darren Hedley

**Affiliations:** 1 Present Address: School of Social and Political Sciences, The University of Melbourne, Melbourne, Australia; 2 School of Psychology and Public Health, Olga Tennison Autism Research Centre, La Trobe University, Melbourne, Australia; 3 Department of Psychology and Counselling, School of Psychology and Public Health, La Trobe University, Bendigo, Australia

**Keywords:** Supported employment, Asd, Job, Work, Vocation, Occupation

## Abstract

The efficacy of the Australian Disability Employment Services (DES) for autistic jobseekers has not been examined and is currently undergoing Government reform. To help inform the new DES strategy, we sought the views of: 24 autistic individuals; seven family members of autistic individuals, and; 46 DES employees. Data were collected using surveys and interviews. Data were analysed using Mann Whitney tests plus deductive thematic analysis based on Nicholas and colleagues’ ecosystems model. Participants highlighted a need to adapt existing policies to enhance flexibility of the DES model. There was participant consensus that DES staff require specific education and training to meet the needs of autistic people. Suggestions to inform the new model of DES for autistic people are made.

## Introduction

The efficacy of the Australian Disability Employment Services (DES) for autistic jobseekers has not yet been examined and is currently undergoing Government reform. To inform the new DES strategy, the views of: 24 autistic individuals; seven family members of autistic individuals and; 46 DES employees were examined. Data were collected using surveys and interviews. Quantitative data were analysed using Mann–Whitney U tests and qualitative data were examined using deductive thematic analysis applying an Ecosystem model. Participants highlighted that the DES could be improved by adapting policies, and by DES staff being trained to work with and meet the needs of autistic people. Suggestions to inform a new DES strategy are discussed.

In Australia, 15% of job candidates with a disability voluntarily seek employment assistance by registering directly with a Disability Employment Service (DES; Australian Government [AG], ( 2022). DES assist people with disability to overcome barriers in finding and maintaining employment (AG Department of Social Services [DSS], (AG Department of Social Services, 2021). Approximately 85% of DES job candidates are registered through Services Australia, the Australian Government agency which provides social and welfare payments to jobseekers (AG, 2022). To receive government financial support, it is typically mandatory for individuals to engage with DES if they are assessed as being capable of working (AG DSS, 2020).

Given concerns over the efficacy of the current DES system, the Australian Government has proposed a reform (AG DSS, 2021). Commencing in 2021, the Australian Government asked key stakeholders’ input into a new model to better serve people with disability, including autistic jobseekers. According to the most recent reports, autistic people comprise 16% of DES providers’ case load, but on average only 25% of them gained employment (AG, 2022). Given DES providers are widely available in Australia and are at no cost to access, they are well placed to support autistic jobseekers into employment.

Autistic people often experience barriers to gaining and maintaining employment (Hayward et al., 2018a); (Wei et al., 2018)) with both under- and unemployment being known issues for this population (Hayward et al., 2018b). The best estimate of the employment rate of Australian autistic adults is 27.3%, significantly lower than adults without a disability (80.3%) as well as those with a disability (47.8%; Australian Bureau of Statistics, 2019)). This is despite autistic adults having attained post-secondary education which is typically indicative of positive employment outcomes (Ohl et al., 2017; Wei et al., 2018).

Little is known about how well DES meet the needs of autistic individuals. It is important to triangulate data from different perspectives to increase its validity and understand how well the DES supports autistic jobseekers. Important perspectives include those of autistic jobseekers, their family, and DES employees who provide the supports.

The current study utilised the Ecosystem model (Nicholas et al., 2018, 2020). This model has been successfully applied in other countries (e.g., Canada) and conceptualises the factors required for the successful employment of autistic people (Klag et al., 2021). It posits that employment opportunities require a team effort from the individual jobseeker, their family, vocational support agencies, actual and potential employers plus co-workers, and the community (Nicholas et al., 2018, 2020). Given the multi-informant perspective offered by the Ecosystem model, it was adopted to examine how well the needs of Australian autistic jobseekers are being met under DES. Doing so might highlight shortfalls that, once known, can help reform the DES. The findings may also inform other employment programs to best meet the needs of autistic jobseekers.

The aim of the current study was to examine efficacy of the Australian DES for autistic jobseekers. A mixed-method study with a concurrent triangulation design was used with the participants encompassing autistic people reporting on themselves as well as some family members regarding their views of the jobseeker working with DES providers. DES employees also shared their perceptions concerning the barriers and enablers to successfully assisting autistic individuals.

## Method

### Research Design

An outline of the components of the Ecosystem model is provided here (Nicholas et al., 2018, 2020). The first component is jobseekers. This encompasses an autistic person’s skills, abilities, insight into their strengths and weaknesses, prior experiences, and how they perceive the world. The next component concerns how well the jobseeker is supported by their family in their pursuit of finding and maintaining employment (Nicholas et al., 2018, 2020). Additionally, the families of autistic people may offer important insight into their abilities because some autistic people experience challenges with self-awareness (Huang et al., 2017; Williams, 2010). Family members also often act as advocates for autistic individuals (Petner-Arrey et al., 2016; Sanderson et al., 2017).

Further supporting jobseekers are skilled vocational providers who assist them with training needs, provide job coaching, and build the capacity of employers to hire and retain jobseekers (Nicholas et al., 2018, 2020). In Australia, these are the same activities provided by DES staff, who include employment Consultants, Business Development staff, and Post-placement Support Coaches (anonymous personal communication, DES manager, December 19, 2019). Consultants work with jobseekers to help them become job ready and find suitable employment. Business Development staff work specifically with employers to help find or negotiate roles for jobseekers. Post-placement Support Coaches work on site with jobseekers once they are placed into a job. However, depending on the size and location of the DES provider, Consultants sometimes perform the latter two roles (i.e., they act as both Consultant and Post-Placement Support Coach).

Employers too support positive employment outcomes for autistic people by being ‘diversity open’ and providing employees with reasonable workplace adjustments (Nicholas et al., 2018, 2020). The final component of the Ecosystem model is community support, including supportive public policy (Nicholas et al., 2018, 2020) such as the policies that support DES operations. Community supports also encompass access to suitable housing, physical and mental health services, leisure activities, transport, and education.

### Participants

The project was approved by the [blinded for review] Human Research Ethics Committee. Following this, the study was advertised to autistic people and their family members via a range of recruitment channels including social media, autism associations, and Technical and Further Education (TAFE) providers in metropolitan and regional areas across Australia. To be eligible to participate in the study, autistic individuals must have used the services of at least one DES provider as a client or prospective client. Similarly, for family members of autistic people, the autistic person the family member was reporting about must have used the services of at least one DES provider as a client or prospective client at least once. Family members could participate without the autistic person they were (anonymously) reporting about also participating in the study. Current involvement with DES was not an eligibility requirement. There was no eligibility requirement for DES employees except for current employment in the industry. Recruitment flyers for DES employees were sent via the newsletter of the DES peak industry body, as well as to individual DES providers.

A summary of participant demographics is provided in Table 1. A total of 84 individuals participated of whom 24 were autistic: 13 women and 11 men (*M age* = 39.88 years, *SD* = 12.29). Seven were parents of autistic people (six mothers; one father; *M age* = 58.43 years, *SD* = 4.86) who provided data by proxy about their (all male) offspring (*M age* = 23.86 years, *SD* = 9.23). Together, this resulted in a total of 31 autistic participants, both self- and parent report (13 women and 18 men; *M age* = 36.26 years; *SD* = 13.38). Finally, 46 DES employees participated; 34 women and 12 men (*M age* = 45.37 years; *SD* = 11.80).

**Table 1:** Participant demographics

	Autistic Person Self Report *n* (%) *N* = 24	Parent Proxy Report *n* (%) *N* = 7	All Autistic People (Self & Parent Report) *n* (%) *N* = 31	DES Employees *n* (%) *N* = 46
Residential/employment location (DES participants only)				
Metropolitan area	8 (33)	3 (50)	11 (39)	21 (46)
Regional area	16 (67)	3 (50)	19 (61)	25 (54)
Highest completed education				
Some secondary school	2 (8)	2 (29)	4 (13)	2 (4)
Completed secondary school	4 (17)	1 (14)	5 (16)	5 (11)
Technical and further education (TAFE)	7 (29)	3 (43)	10 (32)	23 (50)
Undergraduate degree	6 (25)	0 (0)	6 (19)	12 (26)
Post-graduate degree	5 (21)	0 (0)	5 (16)	3 (7)
Other	0	1 (14)	1 (3)	1 (2)
Employment status				
Full-time	0	1 (1)	1 (3)	40 (87)
Part-time	4 (17)	1 (1)	5 (16)	6 (13)
Casual (includes temporary and self-employment)	9 (38)	0 (0)	9 (29)	0 (0)
No paid employment	11 (4)	5 (71)	16 (52)	0 (0)
Years working with/in DES Provider/s M (SD; Range)	5.00 (3.39; 1–15)	4.29 (3.45; 1–11)	4.83 (3.36; 1–15)	6.59 (6.72; < 1–26)
Age at ASD diagnosis M (SD)	30.83 (15.71)	11.00 (5.94)	26.35 (16.35)	N/A
Diagnosed with ASD by *at least one* of				
Psychologist	18 (75%)	5 (71%)	21 (72%)	1 (2%)*
Psychiatrist	2 (8%)	0 (0%)	2 (7%)	0 (0%)
Paediatrician	0 (0%)	3 (43%)	2 (7%)	0 (0%)
Other	0 (0%)	0 (0%)	2 (7%)	0 (0%)
Unsure the type of professional who diagnosed ASD	2 (8%)	0 (0%)	2 (7%)	0 (0%)
All other diagnoses (other than ASD if applicable)				
Anxiety	15 (63%)	4 (57%)	19 (68%)	7 (15%)
Attention hyperactivity deficit disorder	3 (13%)	2 (29%)	5 (19%)	0 (0%)
Bipolar disorder	3 (13%)	0 (0%)	3 (13%)	1 (2%)
Depression	11 (46%)	3 (43%)	14 (52%)	8 (17%)
Intellectual developmental disorder	1 (4%)	0 (0%)	1 (3%)	0 (0%)
Obsessive compulsive disorder	3 (13%)	0 (0%)	3 (13%)	1 (2%)
Personality disorder	1 (4%)	0 (0%)	1 (4%)	1 (2%)
Post-traumatic stress disorder	3 (13%)	0 (0%)	3 (13%)	0 (0%)
Other (e.g., epilepsy)	4 (17%)	2 (29%)	6 (23%)	2 (4%)
No other diagnoses	4 (17%)	2 (29%)	6 (23%)	32 (70%)

We enquired about both sex and gender identity. All participants reported sex and gender as congruent and identified as either male or female

*This person was also autistic

### Autistic People and their Families

Whether via self- or parent-report, all autistic people had a formal diagnosis of Autism Spectrum Disorder (ASD). To confirm autism diagnoses, participants (or their family by proxy) were asked to provide information on the specific ASD diagnosis received, year of diagnosis, and the type of professional who provided it (e.g., psychologist, psychiatrist, etc.), with details provided in Table 1. Most self-reporting autistic participants (58%, *n* = 18) had received an autism diagnosis in adulthood, i.e., ≥ 18 years of age; *M* = 30.83 years, *SD* = 15.71. Those with a parent reporting by proxy received their autism diagnosis in childhood (*M* = 11.00 years, *SD* = 5.94). Including all autistic individuals in this study, the mean age of autism diagnosis was 26.35 years (*SD* = 16.35, range 4–54 years).

Autistic individuals were assisted by DES providers for an average of five years (see Table 1). Approximately half (52%; *n* = 16) were not working at the time of data collection and around a third (32%; *n* = 9) had never been placed into employment by a DES provider (see Table 2). See Table 1 for the employment status of participants at the time of the survey.

**Table 2:** Quantitative DES provider ratings

	Service users			
	Autistic individuals self-report	Parent of an Autistic individual	All service users	DES provider participants (self-ratings)
Has DES past or present helped place you/the jobseeker into employment?	Yes, *n* = 6; 29%	Yes, *n* = 3; 43%	Yes, *n* = 9; 32%	
	No, *n* = 15; 71%	No; *n* = 4; 57%		
			No, *n* = 19; 68%	
Confidence/ability to work with autistic clients	*M* = 1.88; *SD* = 1.17	*M* = 1.83; *SD* = 0.98	*M* = 1.87; *SD* = 1.10	*M* = 5.02; *SD* = 1.42
Understanding of autistic people’s work needs	*M* = 1.89; *SD* = 1.18	*M* = 2.00; *SD* = 1.73	*M* = 1.91; *SD* = 1.28	*M* = 4.53; *SD* = 1.59
Autism knowledge	*M* = 2.22; *SD* = 1.48	*M* = 1.80; *SD* = 1.79	*M* = 2.13; *SD* = 1.52	*M* = 4.39; *SD* = 1.58
Satisfaction rating of past DES providers	*M* = 1.94; *SD* = 1.25	*M* = 2.50; *SD* = 2.35	*M* = 2.09; *SD* = 1.56	*N/A*

Responses were provided on a 7-point Likert scale from 1 (low) to 7 (high)

### DES Employees

The DES employees’ job functions included one or more of Intake and Assessment (*n* = 5, 11%), Consultant (*n* = 21, 46%), Post Placement Support Coach (*n* = 12, 26%), Business Development (*n* = 9, 20%), Manager (*n* = 12, 27%), and other (e.g., Counsellor; *n* = 3, 7%). On average, DES staff had worked in the industry for more than 5 years (*M* = 6.59 years, *SD* = 6.72; range < 1–26 years). Most DES employees worked across two Australian states; Victoria and Tasmania (*n* = 38; 83%). Four (8%) were working in Queensland and two (4%) in South Australia. One (2%) DES participant worked across several states, and one did not reveal their employment location. Few (*n* = 4; 9%) DES employees were employed across both metropolitan and regional areas. Thirty percent (*n* = 14) of DES employees reported having worked with 21 or more autistic jobseekers and 28% (*n* = 13) reported working with less than five (see Table 3).

**Table 3:** Number of Autistic Clients with whom DES staff have worked

Number of Autistic individuals	Previous Autistic clients	Current Autistic clients
Less than 5	*n* = 13; 28%	*n* = 25; 54%
6 to 10	*n* = 9; 20%	*n* = 15; 33%
11 to 20	*n* = 5; 11%	*n* = 3; 7%
21 or more	*n* = 14; 30%	*n* = 1; 2%
Unsure	*n* = 5; 11%	*n* = 2; 4%

#### Materials and Procedure

Prospective participants could choose to take part in a 60-min interview or complete an anonymous online survey hosted on Qualtrics (2017). Both options were offered to participants to respect their communication preferences, as autistic individuals often prefer written communication (Hedley et al., 2018; Howard & Sedgewick, 2021). Interviews were conducted via phone, video conference or face-to-face, depending on participant preference and COVID-19 restrictions at the time. All interview modes were audio recorded preceding verbatim transcription by a professional transcription service. Five autistic individuals, two parents of autistic people and one DES employee participated in an interview (*n* = 8^1^), the remaining respondents completed the survey (*n* = 76).

Quantitative and qualitative questions were uniform within participant groups across both the interview and survey (Table 4). These were developed in consultation with key stakeholders, i.e., autistic individuals, the family members of autistic people, and DES employees. Questions for autistic people and their family focused on how well they believed DES providers understood and met their, or their family member’s, needs as a jobseeker. These two groups (autistic individuals, parent of an autistic individual) will herein be collectively referred to as ‘service users’; however, ‘autistic individual’ and ‘parent of an autistic individual’ will be used when distinction between these two groups is required. Although most questions focussed on one aspect of the Ecosystem model, the vocational provider, some questions were included to highlight other elements of the model (see Table 4).

**Table 4:** Interview and survey questions

Autistic individuals	Family member of an Autistic individual	DES employees
1. Overall, how helpful would you rate the Disability Employment Service (DES) provider/s you've worked with in the past? Where,1 = not at all helpful; 7 = very helpful	1. Overall, how helpful would you rate the DES provider/s your family member has worked with in the past? Where,1 = not at all helpful; 7 = very helpful	1. How confident are you in working with autistic clients? Where 1 = not at all confident; 7 = very confident
2. How confident were you that your past DES provider/s understood your individual workplace support needs? Where, 1 = not at all confident; 7 = very confident	2. How confident were you that your family member's past DES provider/s understood their individual workplace support needs? Where, 1 = not at all confident; 7 = very confident	2. How well do you think you understand the workplace needs of autistic employees? Where 1 = not at all well; 7 = very well
3. How well did your past DES provider/s meet your needs as a jobseeker/employee? Where, 1 = not at all well; 7 = very well	3. How well did your family member's past DES provider/s meet their needs as a jobseeker/employee? Where, 1 = not at all well; 7 = very well	3. Please indicate your knowledge of the presentation of autism in adulthood where 1 = unfamiliar; 7 = very knowledgeable
4. Overall, how satisfied were you with your past DES provider/s? Where, 1 = not at all satisfied; 7 = very satisfied	4. Overall, how satisfied were you with your family member’s past DES provider/s? Where, 1 = not at all satisfied; 7 = very satisfied	4. What are the barriers to successfully placing an autistic client?
5. If our research team could share one message to employers (including supervisors, managers and co-workers) on your behalf, what would it be?	5. If our research team could share one message to employers (including supervisors, managers and co-workers) on your behalf, what would it be?	5. What are the enablers or facilitators to placing an autistic client?
		6. What are the barriers to helping an autistic client maintain employment?
		7. What are the enablers or facilitators to helping an autistic client maintain employment?

For each question, 1 to 4 (1 to 3 for DES employees), all participants were asked, “could you please explain why you provided this rating?”

The questions for DES employees were designed to explore their ability, confidence, as well as the perceived barriers and enablers in assisting autistic jobseekers (see Table 4). All participants were asked to provide a quantitative response to each question on a 7-point Likert scale (1 = *low* to 7 = *high*) before being asked to provide a qualitative explanation for why each of the ratings were given.

To acknowledge participants’ time, survey respondents were offered the chance to participate in a gift card prize draw of AUD$50 for service users and AUD$30 for DES employees. Interview participants were offered a gift card of AUD$40 for service users and AUD$30 for DES employees. It was assumed that DES employees may also be paid for their time by their employer while providing data, hence their incentive to participate was lower than service users.

## Analysis

### Quantitative Analysis

To assist in understanding the efficacy of DES providers in meeting the needs of autistic jobseekers, a quantitative approach was taken to aid objective interpretation of results. Using *IBM’s Statistical Package for the Social Sciences* (SPSS; Version 26), ratings provided on Likert scales by service users and DES employees were compared. The distribution of mean scores on rating scales did not meet assumptions for parametric tests. As traditional transformation did not improve the distributions (Tabachnick & Fidell, 2007), a non-parametric test, a Mann–Whitney U test, was used to compare scores.

### Qualitative Analysis

To examine the quality of support provided to autistic jobseekers, a deductive thematic analysis was undertaken using the Ecosystem framework as a basis for organising responses (consistent with (Nicholas et al., 2018, 2020). As such, theme names were pre-determined by the model; these were: the autistic jobseeker; the autistic person’s family; vocational supports; employers and workplaces; community and infrastructure. Using *NVivo Pro 12,* all participants’ qualitative responses were read three times before deductive thematic coding was undertaken.

Analysis was undertaken by the first author, a PhD qualified person who has published using qualitative methods and undertaken formal study in qualitative methods at a Doctoral level. To ensure rigour, the third author, who has Bachelor (Honours) level qualifications and has published using qualitative methods, checked 100% of the data from each theme in a process of peer debriefing. Inter-rater reliability between both researchers was very high (κ = 0.92); discrepancies between them were discussed before placement of the response conciliated. Given most of the data collected was via an anonymous online survey, member checking was not possible. However, the data were triangulated by consulting three different participant groups, and thick descriptions are provided in the Results section to increase trustworthiness of the data (Denzin, 1989).

## Results

### Quantitative Results

#### Service Users

Likert scale responses in Table 2 indicated that service users rated DES providers low on their: ability to work with autistic clients (*M* = 1.87; *SD* = 1.10); understanding of autistic people’s needs (*M* = 1.91; *SD* = 1.28); and autism knowledge (*M* = 2.13; *SD* = 1.52).

### DES Employees

DES staff rated themselves: above average in their ability to work with autistic clients (*M* = 5.02; *SD* = 1.42); just above average in their understanding of autistic jobseekers’ work needs (*M* = 4.53; *SD* = 1.59); and neither unfamiliar nor very knowledgeable in autism (*M* = 4.39; *SD* = 1.58; see Table 2). Their autism knowledge was most often gained from self-directed learning (63%) and websites (59%; see Table 5).

**Table 5:** DES provider Autism knowledge source

ASD knowledge source (selecting all that supply)	
Myself (i.e., first-hand experience)*	*n* = 18; 39%
Colleagues	*n* = 23; 50%
Friends or family	*n* = 23; 50%
TV or media	*n* = 13; 29%
Social media	*n* = 7; 15%
Self-directed learning	*n* = 29; 63%
Websites	*n* = 27; 59%
Education	*n* = 8; 17%
Other	*n* = 5; 11%

*1 DES employee was also autistic

#### Comparisons Between Service Users and DES Employees

There were significant discrepancies between service users’ ratings of DES providers, and DES employees’ self-ratings on all Likert scales (Table 2). Service users rated DES providers low on ability to work with autistic individuals (*Mdn* = 2.00) compared to DES employees’ self-rating (*Mdn* = 5.00) with a significant difference in scores, *U (*service users: *n* = 23; *n* DES: *n* = 45) = 59.00, *z* = 6.02, *p* < 0.001, and a large effect size; r = 0.73. Further, service users rated DES employees’ understanding of autistic people’s work needs as low (*Mdn* = 1.00) compared to DES staff (*Mdn* = 4.00), *U*(service users: *n* = 23; DES: *n* = 22) = 52.00, *z* = 4.64, *p* < 0.001, with a large effect size; r = 0.69. Service users also rated autism knowledge amongst DES staff as low (*Mdn* = 2.00) compared with DES employees self-report (*Mdn* = 7.00), *U*(service users: *n* = 23; DES *n* = 46) = 63.00, z = 6.00, *p* < 0.001, with a large effect size; r = 0.72.

### Qualitative Results: The Ecosystem Framework

#### The Autistic Jobseeker

When asked about workplace barriers and enablers when working with DES providers, most (55%; *n* = 17) service users acknowledged that individual factors such as social communication, sensory issues, executive functioning, and the experience of stress impacts their employability. For example, the mother of an autistic person explained in an interview that, “anxiety [impacts him at work] if he perceives he has made a mistake, [he] thinks he is going to get fired.” Some autistic survey respondents recognised individual factors as personal challenges. For example, “long hours, noisy workplace[s], stress, social interactions, [and] multitasking [are challenges I have]”; “[I have problems] transitioning between tasks—if I'm focused on one task and another more urgent one comes up, I find it hard to refocus and turn my attention to something new. Prioritising [is difficult].”

Over half (57%; *n* = 26) of DES employees described jobseekers’ individual characteristics as important to understand when assisting autistic people. DES employees stated that jobseeker’s social and communication barriers, sensory issues, lack of prior work experience, and the individual’s ability to manage stress impact their ability to help them find and maintain employment. As the following DES participants reported via survey, employment for an autistic person “… is very dependent on the individual and their specific barriers and nuances. Often [the barriers] can be in relation to social interaction skills.” Or, “there could be a sensory issue, too busy in the workplace, a loud environment, something might trigger anxiety, not understanding social cues, inability to read facial expressions.”

Despite some individual factors being perceived as employment barriers, most (84%; *n* = 26) service users felt that DES staff need to better understand their personal nuances. For example, the following autistic person reflected in an interview:

“… they just try and put me into [work]places, it's like, really? You don't know anything, you're just trying to put me into a [work]place and then it's like, no, actually I can't do that. It's like, yeah, you're trying to get me a job, but it's a job that I can’t possibly succeed at. Like fine, yeah, put me in a place where I'm actually doomed to fail, no that's not right, you can’t put me there.”

DES providers’ understanding of autistic people’s skills, abilities, interests, and preferences were voiced by services users as needing improvement if they are to facilitate positive employment outcomes. The following interview excerpt with an autistic person demonstrates this perceived lack of understanding from DES staff, “So, they've, I guess, recognised my intellect. I don’t know if they've recognised other skills that haven’t been as obvious. I don’t think they've asked me really what my strengths are. So yet again you know, [they show no] initiative.” Likewise, an interview of the father of an autistic person highlights that understanding individual ability is required by DES staff:

“Every meeting [with the DES provider] is a battle. It's not, you know, we've been going to this organisation for close to 18 months and there hasn't been a single positive thing done for [my son], not anything. There has been—sort of high-flying talk, oh here's a possibility, but there's nothing positive to say about it. No potential employers have been suggested, nothing, and so it's just a constant battle to try to push forward. I envy some parents who just throw the Disability Employment Service[s] out the window and they have the talent and skills to go find their own child some employment, because they understand the strengths and weaknesses [of an autistic person] and it's such an effort to work with the Disability Employment Service.”

Most (67%; *n* = 31) DES employees too acknowledged limitations to their understanding of autism and the needs of autistic people when asked to freely respond to, “how confident are you in working with autistic clients?” and “how well do you think you understand the workplace needs of autistic employees?”. Responses appeared hesitant and voiced needing professional training to increase their knowledge or confidence to work with autistic clients, as these separate survey responses suggest:

1. “I am pretty much confident in working with all kinds of people, including ones who have autism. But due to my lack of [autism] knowledge, I would most likely have someone by my side helping me work with the client/s. If there were more clients attending our office with this condition [autism] I would learn more about it, but I mainly know about anxiety and depression because most of our clients have it …”
2. “I am aware to give the person time to answer, the tone to use, the volume and the surrounds they will excel in. I have had excellent results to date with participants with autism in employment but am always aware I need to keep my knowledge up to date and get to know the participant well before proceeding to marketing [them to potential employers].”
3. “My [autism] knowledge is minimal due to the inability to, or lack of access to appropriate and specific training opportunities centred on autism servicing.”

#### The Autistic Person’s Family

Few (10%; *n* = 3) service users, all family members of autistic people, and few (9%; *n* = 4) DES employees identified the importance of family support in the employment process. The family members of autistic people mostly expressed frustration at their involvement not being valued by DES providers, as the following mother of an autistic person states in an interview:

“… I've been given the impression that they don't want pushy parents. I've felt like I'm the pushy parent and, ‘butt out’, sort of thing. That's just a feeling I get. I don't know but, yeah, it's all about Dan* [the autistic jobseeker] and what he wants, and they work with him, they don't work with the parents.” *Name changed to protect privacy

DES employees described family involvement as both a demand and resource, as described below by two DES staff.

1. “A major barrier can also be well meaning parents or support networks. We have to be able to have honest and open communication with them and they need to be realistic about what an employer or DES provider is able to do. For example, we have a…participant with autism who is keen to work in a [details removed for anonymity] position and has stated that he wants to work a couple of days a week—his mother is adamant that we find a role in a [details removed for anonymity] environment as they have activities on other days. When his mother comes to appointments, he agrees with her but when we are able to have discussions with him alone he says he does not want to work in [removed], he likes [removed]. This causes confusion and frustration for the participant [autistic jobseeker] but also makes a disjointed relationship between us and the participant.” (Survey).
2. “ … make sure you get to know the client, talk with the family, find out exactly what their interests are or their passions.” (Interview)

#### Vocational Supports

Most (90%; *n* = 28) service users identified the role of DES as core to the employment success of autistic individuals, as did most (94%; *n* = 43) DES staff. Yet, perspectives differed between stakeholder groups on the supports required. Service users expressed frustration at being unable to obtain the type of employment assistance they feel they need. In an interview, the following autistic person described the lack of skills amongst DES staff to be able to provide adequate assistance:

“… the other problem with DES [consultants] is I have more education than they do. When you write a job application for a teaching job, there’s a lot of selection criteria and it’s quite detailed. I find addressing those selection criteria difficult. But then the DES [staff], they don’t have the skills to help me to write a good application. I’ve put in for loads and loads of jobs and I generally don’t get an interview. So, they don’t have the skills to help me with that.”

Other autistic people mentioned that DES should provide more flexible services, as the following two survey responses illustrate:

1. “They [DES providers] need to be more supportive of alternative methods of getting work, including supporting someone in how to become a freelancer, or self-employed in an area of interest, even if that area of interest is go[ing to] have a longish ramp up time.”
2. “What guarantee could you give to me that you, DES, are actually going to look for suitable roles, employers, work environment, and cater for the individual person on the autism spectrum?”

To cater to employment needs, service users also expressed a desire for DES providers to improve their skill in building relationships with potential employers. This includes building employers’ capacity to offer meaningful work, as the following father of an autistic person described in an interview:

“He [my son] only needs to work seven hours a week or something, it's just a tiny little slot somewhere and it's not a matter of whether you have a suit and tie and shiny black shoes, it's a lot deeper than that. So trying to - and then most of the time the [DES] employment counsellors are reluctant or unwilling to accept the responsibility of trying to [work with an employer to] create a role from scratch like that, so that's a huge battle. Sometimes, we have to go up through layers of management to get them to accept that that's their job and that it's not [my son] looking at job ads that's going to do it, you know?”

Acknowledging their role in helping autistic people find and sustain meaningful employment, DES employees also discussed the importance of, “placing a person in the right position and taking time to make sure it's the right fit.”; “providing one-on-one support if and when required, ongoing training [of the jobseeker], if necessary financial assistance to employers so that they can update or obtain any specialised equipment or tools needed for the client.”; as well as “communication between the employer, the jobseeker, and the Employment Consultant … taking time to make sure it's the right fit.”

#### Employers and Workplaces

Most service users (68%; *n* = 21) reported that their employment success relies on employers being open to diversity, the provision of reasonable adjustments, and recognition of autistic jobseekers’ strengths. However, as the following separate survey responses from three autistic participants demonstrate, these qualities in employers were deemed as lacking:

1. “Boasting about your commitment to disability inclusion, while refusing to hire autistic people, is no better than resume fraud. You [employers] need to accept that most recruitment rituals do little more than rationalise an employer's prejudices, even legitimising them with bogus terms like ‘culture fit’ or ‘emotional intelligence’ … job interviews discriminate against autistic jobseekers … Hiring autistics requires both open-mindedness and humility from employers ….”
2. “Diversity in the workplace can make you [employers] stronger and more adaptive in unpredictable markets, but require a willingness to accommodate differences and do things differently.”
3. “We can make fantastic employees if you [employers] give us a chance and rethink your hiring processes—instead of standard interviews—let us show you what we can do, give us a chance to showcase our skills.”

All (100%; *n* = 46) DES employees agreed it was important to find suitable employers for autistic jobseekers. Yet, having an “… understanding employer …” and “co-workers [who] understand the person's needs” was a reported barrier for them. These DES employees further describe, “the biggest barrier that I have found is breaking through the stigma [with employers] that all autistic clients behave in the same way and are difficult to employ”; “… often the way someone with autism interacts can be seen as odd, and this can make employers worry about the client’s ability to do the role, this is often not a valid concern, but due to clients [employers] not understanding they can make this judgement and it takes a lot for us to educate them about the abilities of the client.”

#### Community and Infrastructure

Overall, 61% (*n* = 19) of service users and 61% (*n* = 28) of DES employees made comments that described community supports as important for the employability of autistic people. This is because these supports assist the individual to learn about their needs and preferences, as the following quote from an autistic person illustrates: “… I have learned [about myself] through personal experience, and with some guidance from my course coordinators.” Community support also assists with positive mental wellbeing; for example autistic individuals felt that “… talking to ‘professionals’ [helps]”; “[I] commit to taking two walks a day, one in the morning and the second after tea. Exercise is important”. Additionally, work preparation can be facilitated by “… going through the college … or a high school or whatever and then going on to do that job preparation.” (DES employee).

The infrastructure and Australian Government policy as it influences DES providers and the way DES work with autistic people was of utmost importance to participants. Service users reflected on current policies that limit person-centred approaches which might otherwise enhance DES’ efficacy. The following interview excerpts from two autistic people provide a more nuanced picture of how they felt current policy impacts DES service provision, reducing it to a ‘tick box’ exercise that negates them being able to obtain needed employment assistance:

1. “They’re [DES providers] just trying to meet their targets. Get you off their caseload. They’re not considering the ramifications of you being placed in a job that I’m going to be miserable in. You’re not really going to enjoy and you’re not going to be very good at … the main thing is that I just don’t think the system works very well, unfortunately. I don’t know why it is the way it is, but I don’t think the system works very well. I think, really, the whole—having all these employment agencies that get paid so much money from the Government, I don’t know if that’s the best system to have. Because they get paid loads of money and some of them don’t do very much, at all.”
2. “So, getting back to the interview at [DES name omitted], what they did, they said were terribly sorry, we can't hook you up to the DES service. However, as a volunteer, if you come over this way, I'll introduce you to—it might have been Marie* [*name changed]- and she's in the mainstream employment section, she can be your case manager. It's like, ‘oh, okay.’ In reality, it was completely useless because it was just like these people struggled to be able to separate me from the people that had signed up to Centrelink [Australian Government agency providing financial welfare support] and are [financially] bound to use the services of an employment agency. Because at [DES name omitted], they would make an appointment every few weeks and you'd go in there. I wanted the assessment—I wanted the “Employable Me^2^”, I wanted them to be like the dating agency, matching me up to an employer. But in reality they were just like your mum. Making sure that you'd done your homework. ‘Have you applied for any jobs?’ You could just see their heart wasn’t in it.”

DES employees also reported feeling hindered by the system within which they operate. When asked how their employer (i.e., DES provider) could improve support to better enable them to assist autistic people, DES employees stated needing greater resources. For example, “provid[e] us with further training, more time to be able to dedicate to those on the spectrum or assist by getting us further assistance with the clients that need that extra attention.”. DES providers suggested the need to “invest, invest, invest—we have great workers who want to learn” which would in turn facilitate positive employment outcomes.

## Discussion

The Australian Government is currently asking for key stakeholder input into the DES system which is undergoing reform (AG DSS, 2021). This study sought to determine if DES providers are currently meeting the needs of autistic jobseekers by considering multiple stakeholder perspectives; consistent with the Ecosystem framework (Nicholas et al., 2018, 2020). Overall, service users felt much improvement is required if DES providers are to effectively assist autistic jobseekers into sustainable employment. However, while there were discrepancies between the opinions of service users and DES employees, all participants agreed that to better meet the needs of Australian autistic jobseekers, industry reform that supports the development of DES staff is required. The viewpoints of stakeholders are discussed as they relate to components of the Ecosystem framework, while also highlighting implications for policy and research.

### The Autistic Jobseeker

This study identified that to meet the needs of autistic jobseekers, DES providers need more support and training to understand autism and its presentation in individuals. For example, providers need more time to dedicate to individual jobseekers to obtain information about their strengths and workplace needs. This finding is consistent with others who report accurate autism knowledge is essential to effectively assist autistic people into meaningful, sustainable employment (Flower et al., 2019; Hedley et al., 2018). Perhaps supporting this finding is that many of the autistic jobseekers in this research had achieved post-secondary qualifications. The lack of such qualifications is typically a barrier to employment (Ohl et al., 2017), so too for people with other disability types (Devine et al., 2021). Yet, most autistic people in this study had not been assisted into employment by a DES provider despite the majority having obtained further education. Considering autistic people represent a relatively small number of DES providers’ current and previous caseload (see Table 3), their limited autism knowledge is perhaps not unexpected. Nonetheless, this highlights the importance of specialised autism knowledge and service availability for autistic people to be considered at a policy level for employment rates to be improved.

Some autistic jobseekers in the current study may have received their autism diagnosis shortly before they commenced working with a DES provider. As such, they may not have been able to effectively communicate their needs because they might not have had a good grasp of these yet. Adulthood diagnoses are increasingly common and can result in autistic adults reconstructing their self-identity which requires time to recognise their needs and strengths (Hickey et al., 2018; Leedham et al., 2020). Additionally, considering autism is a condition marked by challenges/differences in social communication (American Psychiatric Association, 2013), some service users might benefit from having an advocate to help them communicate their workplace needs and strengths (Cope & Remington, 2021; Migliore et al., 2018). Family constitutes an identified key support in disability employment (Nicholas et al., 2017), and may be a valuable resource to DES providers.

### The Autistic Person’s Family

Although both family members of autistic people and DES staff identified the importance of family support in the employment process, it appeared that each party sometimes had a different understanding as to what support entails. Although there was diversity of prior experiences, some family members reported their unique understanding of the autistic jobseeker was not adequately utilised by DES providers. Consequently, some family members felt that resulted in unrealistic understandings of the capacities of their child, particularly where the autistic person had limited self-insight into their own capabilities. The data suggested that family support may need to be negotiated between the vocational provider and jobseeker. It is important that all parties have a clear understanding of their roles and boundaries, keeping in mind there will be different capabilities of support networks to assist in the process. In other studies of people with disability, it has been found that family support increases the likelihood of economic participation (Carter et al., 2017; Hetherington et al., 2010)). Thus, it is suggested that with jobseeker consent, triangulating information with at least one person in the jobseeker’s support network could be part of procedural change.

### Vocational Supports

Despite DES employee’s quantitative data indicating better than average ratings on their ability to work with autistic clients, and a slightly better than average understanding of their work needs, their qualitative data suggested differently. Most DES employees recognised they need further support to adequately assist autistic jobseekers. Some acknowledged limitations to their understanding of autism as well as the needs of autistic people. The high rate of autism inexperience in the DES employee group could be due to the relative proportion of participants in management positions (27%), as these individuals may have little to no direct jobseeker contact. Supporting this, 28% reported having worked with less than five autistic jobseekers. However, a similar proportion reported having worked with 21 or more. Lack of support could be the reason most DES employees stated gaining their autism knowledge via self-directed learning. It is also possible that DES staff may have difficulty evaluating the accuracy of the information they obtain, especially via self-directed learning.

Other research has found that vocational support staff are more likely to perceive themselves favourably compared to service users (Nicholas et al., 2017). This could help explain the large discrepancies between DES employee self-ratings and service user ratings of DES. It is noteworthy that current policies expect DES providers to achieve employment outcomes for people with all disability types equally well (AG DSS, 2021). Alternatively, there may be measurement error in this study resulting in partial acquiescence response bias to quantitative questions (Sauro & Lewis, 2016).

This research highlights that people supporting autistic jobseekers, such as DES staff, need access to accurate disability specific knowledge and the ability to apply this knowledge to help their client secure sustainable employment. This will also enable DES employees to educate employers about autism and negotiate the adjustments needed for autistic jobseekers. Elevating the training and educational requirements for DES staff as part of service provision policy would assist in achieving successful employment outcomes for autistic jobseekers.

### Employers and Workplaces

Black et al. (2019) and (Hurley-Hanson et al., 2020) reported that working with employers can be challenging, particularly those who possess inaccurate information about autism. Both service users and DES employees described lack of employer openness to diversity as a barrier to the employment of autistic jobseekers. This may be overcome if employers have access to external support to hire and retain autistic individuals (Rashid et al., 2017, 2018). For example, an autism specific vocational provider to support the employer. There is a need for greater awareness regarding diversity in Australian organisations so employers understand the value of diversity in all its forms, including neurodiversity (see, Davis et al., 2016; Hayward et al., 2018a, 2019) Thus, it is suggested that the benefits of diversity, as they relate to organisational outcomes, should be widely promoted facilitated via Government channels.

### Community and Infrastructure

Both service users and DES employees reported that incentivising job-placements over job-fit to receive Australian Government funding can negatively influence DES providers, hindering successful sustainable employment of autistic people. Service users further supported the idea that because DES providers must meet Government requirements, this reduces assistance to a ‘tick box’ activity and restricts their ability to provide effective services. Therefore, the ability of vocational providers to work with autistic jobseekers and achieve the job-person-environment-fit required for employment success remains a significant challenge (Nagib & Wilton, 2019; Nicholas et al., 2019). Focusing on sustainable outcomes as a measure of DES success rather than job placements alone may improve the current system for autistic job seekers. This may be achieved by adoption of a non-time limited customised employment approach (Wehman et al., 2016).

A successful evidence-based Customised Employment model is available through vocational providers in the United States and is a ‘one stop shop’ (Wehman et al., 2016). Noting also that in Australia some DES staff have one specific role before the jobseeker is transitioned to another DES staff member for further support (anonymous personal communication, DES manager, December 19, 2019), better outcomes might be achieved if a single DES employee works with the autistic jobseeker for the entire employment journey.

Considering variability in DES employee skill, ability, and experience, it could be useful if autistic jobseekers are given the necessary information and opportunity to select a vocational provider. Giving service users this type of choice and control also aligns with the *Australian National Disability Insurance Scheme Act* (2013) which suggests that jobseekers should be able to choose where and how they receive assistance (see Sect. 3.1.e of the Act).

Further, participants in this study recognised the experience of jobseeker stress as negatively impacting employability; thus, employment assistance could include appropriate access to or inclusion of mental health support both while looking for and during employment. The importance of access to mental health services for autistic people has been previously recognised (Bury et al., 2022; Hayward et al., 2020).

### Limitations

Several study limitations need to be acknowledged. First, given the high unemployment rate among autistic individuals in this study (52%), participants may have been more likely to rate DES providers negatively. This finding is similar to (Devine et al., 2021) who reported that unemployed individuals experienced more employment barriers and wished for more vocational provider support. Notwithstanding this, approximately 25% of all data were collected during the first wave of the COVID-19 pandemic which may also have impacted reported employment rates and subsequent ratings of DES providers. However, only a third of autistic jobseekers in this research had ever gained employment via a DES provider (see Table 2). However, the relatively small sample limits generalisability of the results.

Second, service users and DES providers were not matched in this research; all observations were independent. Future research would benefit from matched observations with a large sample to aid the ability to compare groups more fairly. Comparisons might also be considered between the perspectives of those in metropolitan and regional areas whose employment support needs may differ.

Finally, given the parents who provided proxy accounts in this study reported receipt of an autism diagnosis at a younger age for their child, those who receive an autism diagnosis at a younger age may have differing support needs. This was not explored in this study and warrants further investigation.

## Conclusion

To better meet the needs of Australian autistic jobseekers, service users and DES employees agree that industry reform that results in greater support for DES staff is required. To aid the Australian Government in reforming the DES, six suggestions are made based on the findings from the current study: (1) vocational providers and/or individual staff members within these could specialise in disability type where training and support is provided via reputable sources; (2) flexibility should be incorporated into vocational support operations so autistic jobseekers can choose a vocational provider. Enhanced training and flexibility within the system need to be conducive to a person-centred, tailored, or customisable approach which focuses on individually derived outcomes at a pace relative to the individual; (3) vocational providers need to consider a holistic approach to supporting jobseekers and include people in their support system where appropriate and possible; (4) focus on systems that reward vocational providers for sustainable employment outcomes; (5) provide training and support to vocational providers so they are able to work more effectively with potential employers; (6) work with employers at a community level to encourage diversity open organisational climates.

## Footnotes

1 Demographic data are not provided separately for the participants who completed an interview to ensure these individuals cannot be identified.

2 A reality television show which showed a customised employment process tailored to the individuals.
